# Unusual dermatomycoses caused by *Nannizzia nana*: the geophilic origin of human infections

**DOI:** 10.1007/s15010-020-01416-5

**Published:** 2020-03-30

**Authors:** Sebastian Gnat, Dominik Łagowski, Aneta Nowakiewicz, Mariusz Dyląg

**Affiliations:** 1 Department of Veterinary Microbiology, Faculty of Veterinary Medicine, Institute of Biological Bases of Animal Diseases, University of Life Sciences in Lublin, Akademicka 12, 20-033 Lublin, Poland; 2 Department of Mycology and Genetics, Faculty of Biological Sciences, Institute of Genetics and Microbiology, University of Wroclaw, Przybyszewskiego 63/77, 51-148 Wroclaw, Poland

**Keywords:** *Nannizzia nana*, Onychomycosis, Superficial infection, Diagnostics, Genetic diversity

## Abstract

**Background:**

Fungal infections of the skin, hair, and nails are the largest and most widespread group of all mycoses. *Nannizzia nana* is a relatively rare etiological factor of dermatomycosis in humans, as it usually affects animals, e.g. pigs and boars. In addition to the zoophilic nature, there are also reports of the geophilic reservoir of this dermatophyte species.

**Objective:**

In this study, we present symptomatic infections with *N. nana* aetiology in humans reported recently in Poland. Interestingly, these cases had a non-specific clinical picture and occurred as skin lesions on the neck and foot as well as onychomycosis of the toenails. From the medical history, the patients had no contact with pigs.

**Methods:**

Diagnostics of these infections was performed with a combination of classical phenotypic and molecular genomic methods. The genomic diversity of the isolates was determined using the MP-PCR method. In vitro antifungal susceptibility tests against itraconazole, ketoconazole, terbinafine and naftifine hydrochloride were also performed.

**Results:**

*Nannizzia nana* has been identified as an etiological factor of dermatomycosis. Moreover, heterogeneity of the genomes was revealed for the obtained strains. In vitro activities of antifungal agents showed that isolates were susceptible to all tested drugs. The patients were treated with oral terbinafine and topical ketoconazole cream, which led to a complete recovery.

**Conclusions:**

In conclusion, the cases studied by us may indicate that the infrequency of *N. nana* infections may not necessarily be related to the low infectivity of this fungal agent, but they are rather associated with misdiagnosis. Furthermore, *N. nana* reservoirs should also be sought in soil.

## Introduction

Dermatophytes are keratinophilic fungi that affect nails, hairs, and skin of humans, warm-blooded animals, and others [1, 2]. Approximately, 20–25% of the global human population is infected with a dermatophyte at least once per lifetime [3, 4]. About 30 clinically relevant dermatophyte species are known, but their taxonomy has been controversial because of the incongruence of phenotypic and molecular characters [5, 6].

The genus *Nannizzia* was introduced by Stockdale [7] with *Nannizzia incurvata* Stockdale 1961 as a type species to accommodate *Microsporum*-like species producing gymnothecia, which were discovered in 1927 by Nannizzi. Most of the species classified in the genus *Nannizzia* were described with double nomenclature after finding their heterothallic sexual form [1, 8]. Currently, for practical reasons and necessity, de Hoog et al. [6] proposed the sexual names as nomenclatural reference with molecular differentiation as the leading classificatory principle. *Nannizzia* (likewise the genus *Arthroderma*) is separated as an independent, holomorphic genus located between *Trichophyton* and the preponderantly zoophilic genus *Microsporum *[6]. Several species were found to cluster in the well-demarcated *Nannizzia* group, e.g. *N. nana* (C.A. Fuentes) Y. Gräser and de Hoog (2016) [6], formerly known as *Microsporum nanum* C.A. Fuentes (1956)[9].

*Nannizzia nana* is the common cause of ringworm in the pig [4, 10]. In the 80s, approximately 27% of pigs were infected by *N. nana*, since these infections were easily transmitted between animals in the same flock [10]. In recent years, there are scarce reports on the occurrence of infections caused by this dermatophyte species. In 2009, an outbreak of ringworm caused by *N. nana* was reported in sows in Spain [11]. In exceptional cases, infections have been reported in other animals such as dogs, cats, and mice [12–14]. It is also important to underline that this dermatophyte species is usually acquired directly from the soil, rather than from other animal hosts [4, 14]. *N. nana* is distributed worldwide, but only few cases have been described in humans [11, 12, 15].

The aim of our study was to carry out diagnostic analysis to confirm species identification of *Nannizzia nana* isolates. All the strains were isolated from humans with clinical symptoms of dermatomycosis and identified using conventional laboratory methods, ITS sequencing, MP-PCR differentiation, and antifungal susceptibility testing.

## Materials and methods

### Dermatophyte strains

**Fig. 1:**
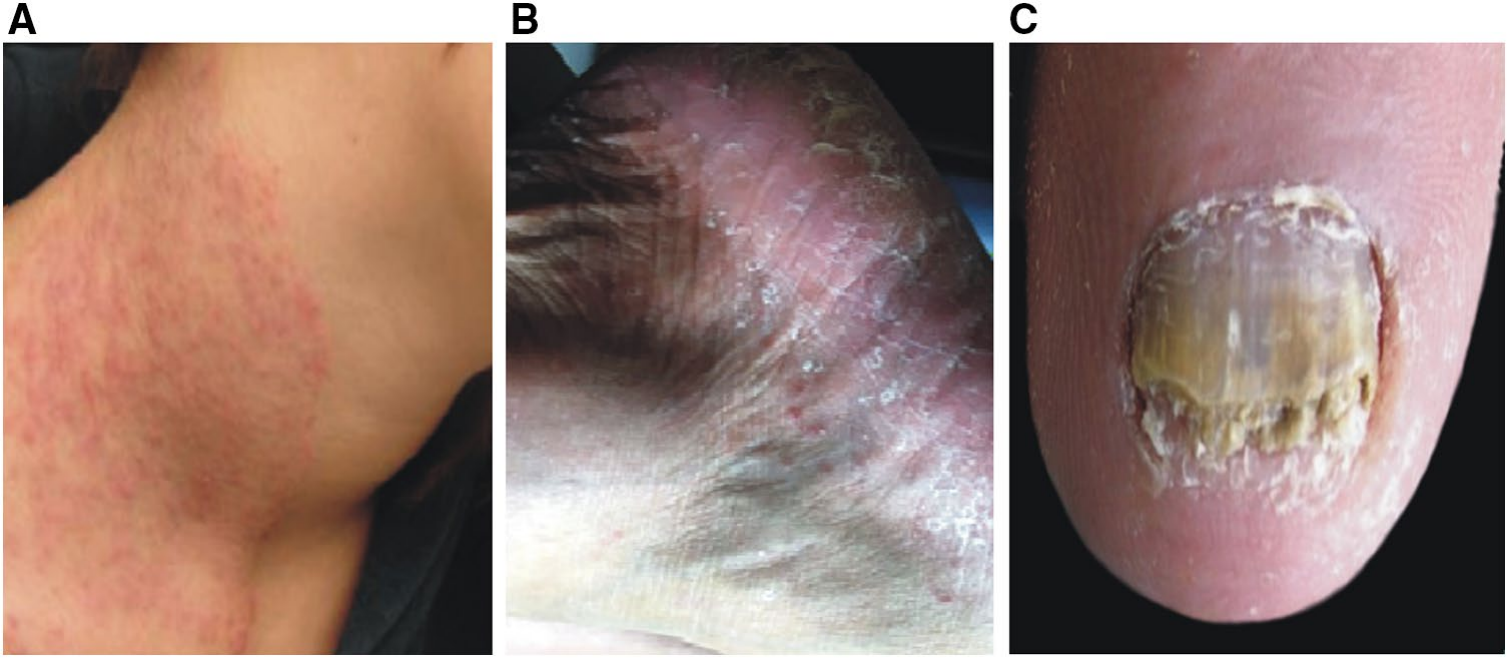
Changes in human skin during infection with *Nannizzia nana* (camera: Nikon D3300, lens Nikon 18–105 mm VR). **a** In the man; **b** in the 41-year-old woman; **c** in the 75-year-old woman

The dermatophyte isolates used in this study were obtained from three clinical cases. The first was a 28-year-old male resident of an urban area in central Poland, who attended dermatological consultation due to 20-day localized superficial skin lesion on the neck (Fig. 1a). The clinical lesion was erythematous and scaly, sharply demarcated, with active borders, and although no chronic scratching marks were seen, the patient reported itching. The man had not suffered from superficial mycosis earlier and had no other chronic diseases. As shown by his medical history, the patient does not breed pets and has not been in contact with objects of animal hygiene recently. However, the man had contact with the soil while carrying out works in the allotment garden.

The second case was a 41-year-old female resident of a rural area in eastern Poland. The patient came for dermatological consultation with 1-year long-standing skin lesions. Clinical changes included erythematous squamous plaques on both feet (Fig. 1b). The woman reported severe itching with a burning sensation. The patient used ointment with terbinafine for 2 months before seeking medical advice. The medical history indicated that she did not suffer from other illnesses and did not take any medicines on a long-term basis. The woman professionally deals with cultivation of the field and growing vegetables in a small farm. The woman is in constant contact with animals, mainly cattle and two dogs.

The third case was diagnosed in a 75-year-old female resident from a rural area of eastern Poland, who asked for medical advice because of recurrent onychomycosis (Fig. 1c). The lesions occurred for the first time about 7 months earlier. Clinical changes affected the nail plate, which was grey, matte, and crumbling. During this period, the patient did not use any antifungal agents. The woman suffers from geriatric problems and is under the supervision of an internist. As shown by the interview, the woman is an owner of three cats who are often away from the homestead.

All three patients received treatment with terbinafine orally at a dose of 250 mg/day and topically with ketoconazole 2% cream applied twice daily to the affected areas. In the case of the man, the treatment lasted 20 days; in the second case described above, 40-day treatment was administered. The woman with onychomycosis was advised to remove the nails and was treated for 6 weeks. In all the three cases, the treatment was fully successful, with complete clearance of the lesions. None of the patients experienced a relapse during the next few months of the follow-up period. The eradication of the infection in the case of all the patients was evidenced by negative direct microscopy and culture.

### Laboratory diagnostic procedures

Species identification of the isolates were performed with a combination of classical phenotypic and molecular genomic methods as described previously by Gnat et al. [16, 17]. In brief, direct examination of the clinical material collected from the patients, i.e. skin and nail scrapings treated with dimethyl sulphoxide (DMSO) and 10% KOH, was performed. Each time the diagnostic material was inoculated simultaneously onto Sabouraud’s glucose agar (Becton Dickinson, New Jersey, USA) at 37 °C for 3 weeks and Dermatophyte Test Medium (BioMaxima, Lublin, Poland) at 28 °C for 2 weeks. The fungi were identified based on colony texture, production of typical mycelium structures, especially species-specific macroconidia, and positive reaction observed on the DTM. Genomic DNA was isolated from the cultures of dermatophytes according to the phenol–chloroform method [18]. Molecular identification was performed by Internal Transcribed Spacer (ITS) region amplification and PCR product sequencing with primers ITS1/ITS4 [19]. The melting profile PCR (MP-PCR) method was used to determine the genomic differentiation of the isolates [20].

### Antifungal susceptibility testing

Since a number of recent reports have revealed emergent drug resistance among clinical isolates of dermatophytes, in vitro antifungal susceptibility tests against itraconazole, ketoconazole, terbinafine and naftifine hydrochloride were performed in this study. All the compounds used in the present experiments were, if not stated otherwise, purchased from Sigma-Aldrich (Missouri, USA) and were of analytical grade. Susceptibility assays were performed according to the Clinical and Laboratory Standard Institute (CLSI) M38-A2 document [21]. All analyses were made in triplicate.

## Results

**Fig. 2:**
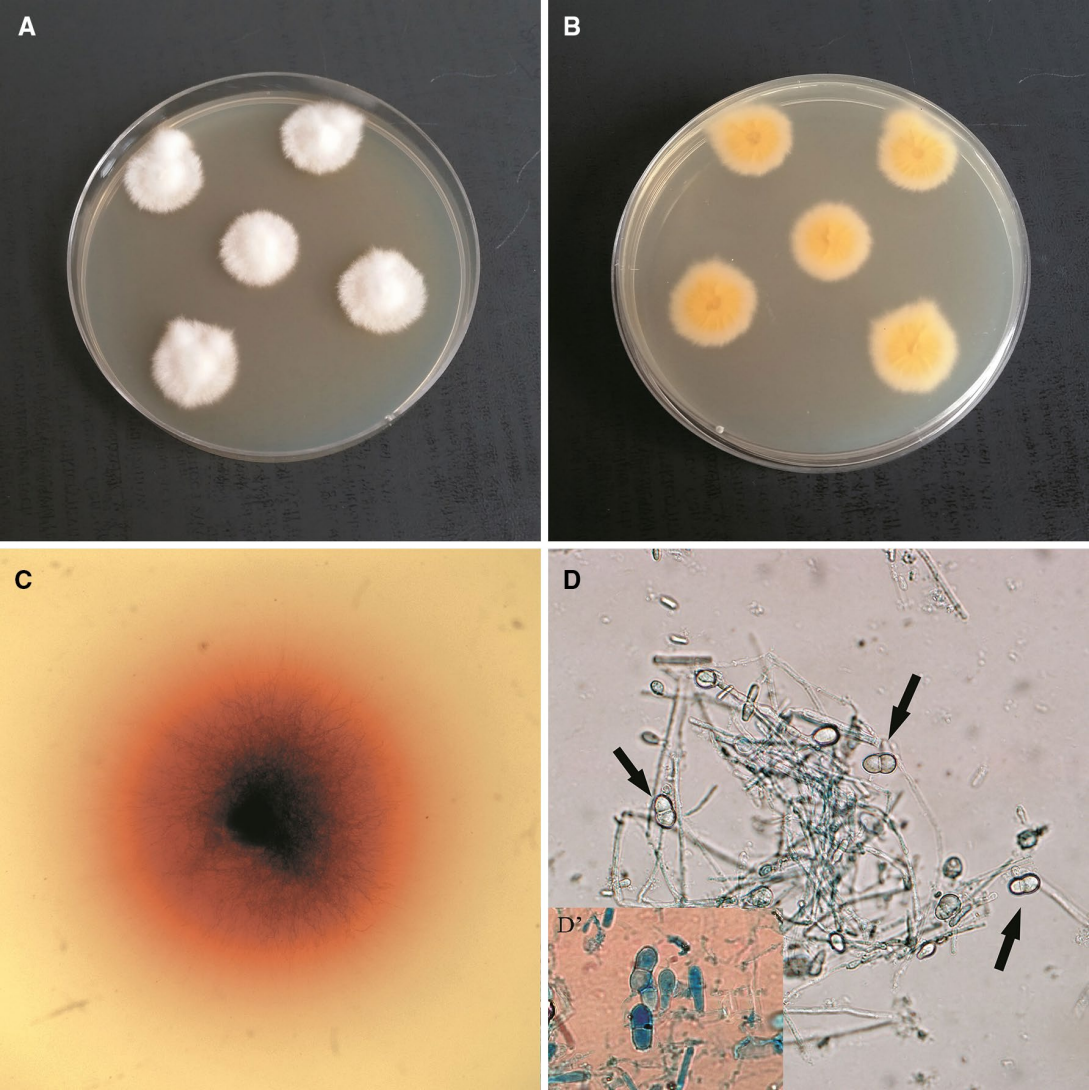
Micro- and macroscopic morphology of isolated dermatophytes, Nikon Coolpix YS100). **a** Obverse of *Nannizzia nana* isolate; **b** reverse of *Nannizzia nana* isolate; **c** positive reaction on Dermatophyte Test Medium; **d** micromorphology, arrows indicate characteristic macroconidia (magnification 400×); **d**’ macroconidia stained with lactophenol blue (magnification 1000×)

**Fig. 3:**
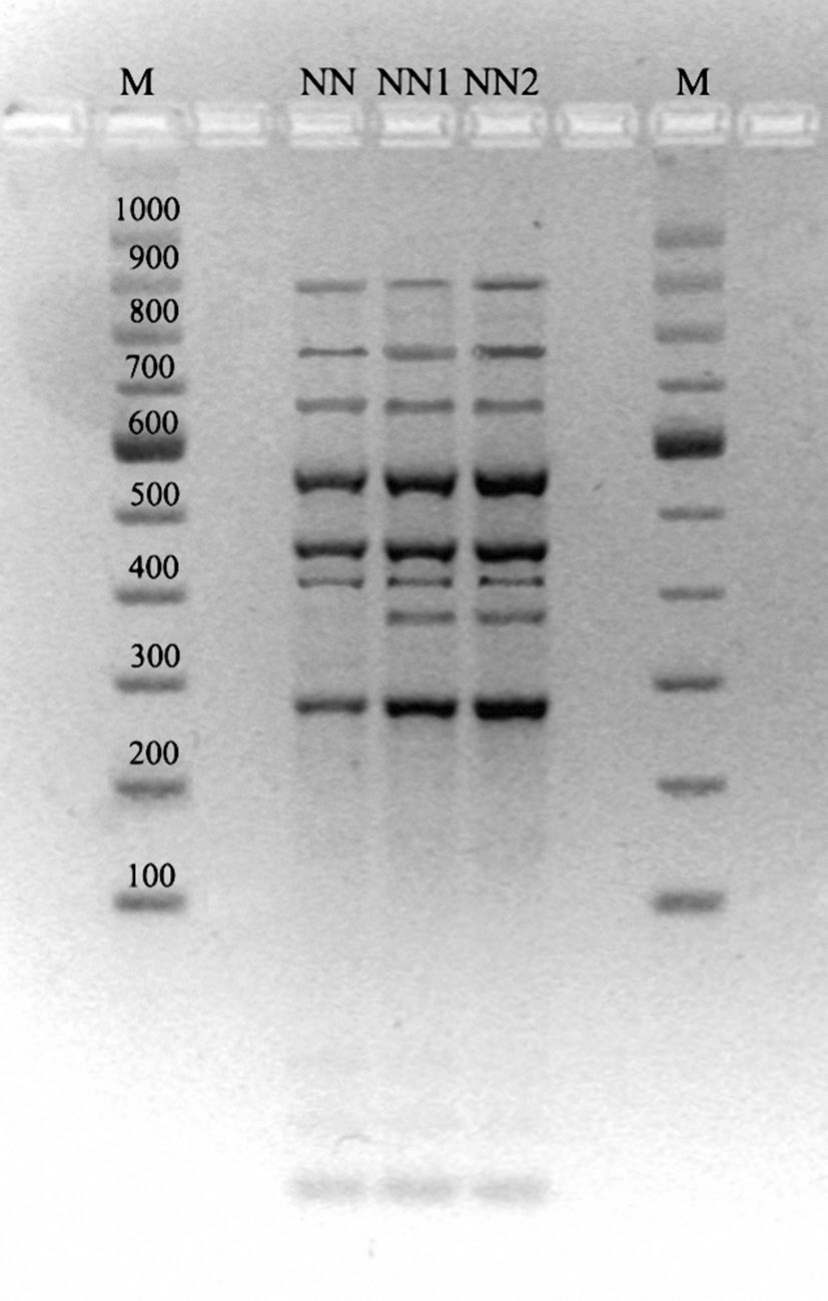
Electrophoretic profile obtained with MP-PCR fingerprinting methods in 3% agarose gel. M-Molecular weight marker A&A Biotechnology (100–1000 bp), NN—strain isolated from the man, NN1—strain isolated from the 41-year-old woman, NN2—strain isolated from the 75-year-old woman

The direct analysis of the material revealed the presence of arthrospores in the samples collected from the skin lesions. The macro- and micromorphology of the colonies and mycelial structures obtained from the clinical material of the three patients were almost identical and suggested that all the strains belong to one specific species (Fig. 2). After 3 weeks of incubation, the colonies were gently fluffy and cream to beige in colour with a suede-like texture; reddish-brown pigmentation was present on the reverse side of the colony. Microscopic examination of the culture-derived preparations stained with lactophenol cotton blue (LPCB) revealed short pyriform thick-walled macroconidia with one to three (mostly two) cells. Based on the morphology and classical phenotypic methods, all the three dermatophyte isolates were identified as *Nannizzia nana.* A comparative analysis of ITS sequences (PCR products obtained with ITS1 and ITS4 primers) of the isolated strains with the sequences of reference strains available in the NCBI (National Center for Biotechnology Information) database revealed a 99% similarity to *Nannizzia nana* CBS 314.54 (Table 1). The MP-PCR showed genomic diversity of the examined dermatophyte strains. The examination based on agarose gel electrophoregram indicated two different types of profile: one characteristic for the strain isolated from the man and the other one specific for the clinical strains obtained from both women (Fig. 3). Screening of the material taken from animals with which the women had contact showed no presence of the dermatophytes.

**Table 1:** Isolates of dermatophytes with description

Isolates	Host	Location of changes	Accession numbers of ITS sequences	Identification consistent with the NCBI database	Drug sensitivity, MIC values [µg/ml]			
					Itraconazole	Ketoconazole	Terbinafine	Naftifine
NN	Human	Neck	MN307390	*Nannizzia nana* CBS365.53 (accession number NR154982.1) 99%	0.5	0.125	0.125	0.25
NN1	Human	Foot	MN307391		0.25	0.125	0.25	0.5
NN2	Human	Nail	MN307392		0.5	0.25	0.25	0.5

*NCBI* National Center for Biotechnology Information, *MIC* minimal inhibitory concentration

In vitro activities of antifungal agents that can potentially be used either orally or topically showed that all the isolates tested were susceptible to itraconazole, ketoconazole, terbinafine, and naftifine hydrochloride (Table 1). Minimal inhibitory concentration (MIC) values among the isolates varied from 0.125 to 0.5 µg/ml. Geometric means of the minimal inhibitory concentration of ketoconazole (0.167 µg/ml) and terbinafine (0.208 µg/ml) were the lowest for the examined isolates, indicating that these drugs were the most potent in the therapy of each of the cases described in this work. Nonetheless, the mean MICs of the antifungal drugs did not exhibit statistically significant differences between each other (*p* > 0.05).

## Discussion

*Nannizzia nana*, reported previously as *Microsporum nanum*, was first described in 1954 by Fuentes et al. [9] in Cuba as the aetiological agent of tinea capitis in a child, identified as a dwarf form of *Microsporum gypseum* [22]. Swine are the natural host for this dermatophyte [22]. *N. nana* is regarded as a low-virulence aetiological agent; it was even thought to be part of pigs’ skin microbiota [23]. The majority of human patients with dermatomycosis caused by *N. nana* was linked with direct daily or long-lasting contact with these animals on pig farms [15, 22, 24, 25].

Clinical pictures of *N. nana* infections in humans are related mainly to tinea corporis with a characteristic ring shape with an erythematous, scaly, circinate plaque and, rarely, dry or inflammatory tinea capitis [11]. Occasionally, lesions on the skin may suggest *Microsporum canis* infection [15]. Bonifaz et al. [22] described two severe cases of *N. nana* infection in siblings in 2019, in which an 8-year-old boy was affected by mycosis of the scalp developing as a pseudoalopecic tumour lesion and a 6-year old girl, his sister, presented with dermatomycosis characterized by multiple erythematous-scaly plaques on her face, trunk, and arms. Dermatomycosis among children in Mexico is closely related to the coexistence with pigs on the same farm [22]. The authors of these publications do not consider other sources of *N. nana* infection in these two cases.

Our study reveals, however, completely different characteristics of this dermatophyte species. The three reported cases occurred in Poland in the summer months of 2018 in patients living at a distance of about 150 kms from each other. None of them reported contact with pigs. In addition, the location of the clinical lesions, which covered the edge of the foot and toenails in two of the three described cases, did not indicate a zoonotic origin of the dermatomycosis and might suggest rather a geophilic source. In the literature, there are also reports on exceptional cases of tinea pedis and onychomycosis caused by *N. nana* [26, 27]. Furthermore, *N. nana* reservoirs should also be sought in soil, and the information on the dual nature of this dermatophyte, both zoophilic and geophilic, seems to be relevant [28].

Interestingly, genotyping of clinical isolates by MP-PCR showed no homology of the strains. Two different types of profile were obtained: the first one characteristic for the male patient and the other one determined for the two female patients. Finding causal links between this genomic similarity and infection epidemiology can only be speculative, but it is important that methods for determining genomic diversity can also be used for *N. nana*. There are no reports in the literature about genomic polymorphism in this species of dermatophyte. In many outbreaks of dermatomycoses of different aetiology, e.g. caused by *Microsporum canis* [17, 29, 30] or *Trichophyton verrucosum* [20], genotyping methods have been found greatly suitable in searching for the most probable sources of infection and determining pathogen transmission pathways. Comprehensive analysis on this issue for *N. nana* infections is necessary to reach definitive findings.

Unfortunately, in the literature, there are only few reports on the minimal inhibitory concentration (MIC) values of *N. nana*. Hence, antifungal therapy is usually chosen based on the response observed when treating other dermatomycoses caused by fungi of the genus *Microsporum* [22, 31]. In our study, the in vitro susceptibility to the main antifungals indicates that *N. nana* is highly sensitive to itraconazole, ketoconazole, terbinafine, and naftifine hydrochloride. Wildfeuer et al. [32] observed the following MICs for *N. nana* (*Microsporum nanum* in the original publication): griseofulvin 3.1 μg/ml; voriconazole and itraconazole 0.78 μg/ml, and ketoconazole 0.2 μg/ml. In turn, in their case report of dermatomycoses in the siblings, Bonifaz et al. [22] noted that *N. nana* was sensitive to miconazole, clotrimazole, and ketoconazole without specifying the inhibitory concentration range of these drugs. Furthermore, there are no precise data in the literature about *N. nana* sensitivity toward terbinafine and naftifine hydrochloride*.* Noteworthy, these studies include only few strains. This issue requires more extensive research and deeper discussion.

Dermatomycoses are still often misunderstood and underestimated. The infrequency of infections caused by many species of dermatophytes, including *N. nana*, may not be related to the low infectivity of this fungal agent, but rather to misdiagnosis. Differential diagnostics based on classical and molecular methods is equally important as the knowledge of the sources and reservoirs of dermatophytes. As emphasised by many experts, these are the keys to proper therapy.
